# Environmental Exposures and Human Diseases: Molecular Insights

**DOI:** 10.3390/biomedicines13112830

**Published:** 2025-11-20

**Authors:** Maaike van Gerwen, Luoping Zhang, Angela M. Leung

**Affiliations:** 1 Department of Otolaryngology-Head and Neck Surgery, Icahn School of Medicine at Mount Sinai, New York, NY 10029, USA; 2 Division of Environmental Health Sciences, School of Public Health, University of California, Berkeley, CA 94720, USA; luoping@berkeley.edu; 3 Division of Endocrinology, Diabetes, and Metabolism, Department of Medicine, David Geffen School of Medicine at University of California Los Angeles, Los Angeles, CA 90095, USA; amleung@mednet.ucla.edu

Environmental exposures play an important role in the development of diseases [1]. Around the world, populations are exposed to rising levels of environmental pollutants, due to increasing transportation and industrial emissions, agricultural runoff, and fossil fuel combustion, leading to increased pollution-related deaths [2]. Pollution is currently the second-leading cause of non-communicable diseases, leading to rising rates of cardiovascular, respiratory, and neurodegenerative diseases, as well as cancers [3]. This highlights the importance of understanding these environmental risk factors and elucidating the pathways that induce these negative health effects.

Throughout life, environmental exposures affect the human body, directly impacting barrier organs such as the lungs, skin, or gut and indirectly impacting other organs, for instance, through systemic responses. Peters et al. proposed eight hallmarks of environmental insult to describe potential pathways linking exposures to chronic diseases: (1) oxidative stress and inflammation; (2) genomic alterations and mutations; (3) epigenetic alterations; (4) mitochondrial dysfunction; (5) endocrine disruption; (6) altered intercellular communication; (7) altered microbiome communities; (8) impaired nervous system function [4]. This framework shows that chronic exposure to modest concentrations of pollutants, particularly exposures that can impair multiple pathways, may induce severe health effects.

Following this framework, this Special Issue focuses on providing molecular/cellular insights into the impacts of a variety of environmental exposures on human diseases and presenting the latest advances in the field of environmental medicine.

Shvachiy et al. investigated the sex-specific effects of intermittent and chronic low-level lead exposures on the cardiorespiratory system and neurotoxicity [5]. As there are no safe levels of lead exposure, the investigators were particularly interested in the potential sex differences in the impacts of this exposure in a rodent model. They found stronger health effects, including hypertension, anxiety, and reactive astrogliosis, following permanent exposure compared to intermittent exposure, independent of sex. Males, however, were more susceptible to cognitive, behavioral, and respiratory changes, while females were more prone to chemoreflex hypersensitivity. This confirmed that, in order to glean a better understanding of the impact of an exposure on health, not only the type (chronic versus intermittent) of exposure but also the sex of the exposed individual is relevant. A comprehensive understanding of sex-related differences in toxicity will enhance personalized environmental medicine.

Tebbi et al. present current evidence on the potential role of the mycovirus-containing *Aspergillus flavus* in acute leukemia [6]. Virus-containing *Aspergillus flavus* can alter the genetics of human cells. Furthermore, patients with acute lymphoblastic leukemia have antibodies against this organism. Following the current literature, the authors hypothesized that environmental exposure to fungi harboring mycoviruses may lead to genetic alterations and dysregulation of key transcription factors, particularly in people with inherited or acquired predispositions and immune system vulnerabilities. This will then lead to the accumulation of genetic and epigenetics changes and the development of acute leukemia. This review describes the accumulation of multiple environmental insults in the pathway from exposure to the development of leukemia.

Stein summarized the literature on bisphenol A (BPA), an endocrine-disrupting chemical, and certain human diseases, including autism spectrum disorders (ASDs), attention-deficit disorder (ADD), Parkinson’s disease (PD), polycystic ovary syndrome (PCOS), and Alzheimer’s disease (AD) [7]. BPA glucuronidation efficiency and disease can be explained through a direct and indirect pathway. Following the direct pathway theory, BPA is the causative agent; compromised BPA detoxification leads to higher BPA concentrations and thus the exposure of organs to its endocrine-disruptive effects. The indirect pathway theory suggests that BPA is a marker for decreased glucuronidation efficiency of another unknown compound that acts as an endocrine disruptor, leading to a metabolic environment favoring disease development.

Cyna et al. summarized the literature on environmental factors and the development of auto-immune thyroiditis and found a variety of different factors of importance, including iodine intake, vitamin D deficiency, selenium deficiency, viral infections caused by Epstein–Barr virus (EBV), human parvovirus B19 (PVB19), human herpesvirus 6A (HHV-6A), and severe acute respiratory syndrome coronavirus 2 (SARS-CoV-2), bacterial infection caused by Helicobacter pylori, microbiome disruption, and medications such as interferon-alpha and tyrosine kinase inhibitors, as well as stress, climate factors, and smoking [8]. This highlights the importance of diet and lifestyle in a patient’s assessment.

Rosenfeld explored the potential harmful effects of endocrine-disrupting chemicals (EDCs) on unborn children from a prevention perspective and summarized the literature on the usage of maternal probiotic supplementation to mitigate exposure to endocrine-disrupting chemicals and thus prevent potential associated negative health impacts in unborn children, focusing in particular on neurobehavioral and metabolic disorders [9]. To date, studies suggest that probiotics during pregnancy may improve both maternal health and health outcomes in offspring. However, no study has directly investigated whether maternal probiotic supplementation combats EDC exposure in pregnant women and their unborn offspring.

Yeshoua et al. presented a review of the association between exposure to flame retardants and thyroid function, including thyroid cancer, in humans [10]. The 61 studies included show that the impacts of various flame retardants on the thyroid are very heterogeneous and dependent on the type of flame retardant and the thyroid function investigated. However, these impacts may have serious consequences, in particular for at-risk populations, and future research should investigate the molecular mechanisms associated with this exposure.

Based on the results of the studies included in this Special Issue, important concerns to address in future research include the investigation of sex-specific impacts, understanding potential cumulative insults, interaction with other (known) risk factors, and heterogeneity within certain classes of pollutants. In particular, a better understanding of the biological/mechanistic/molecular effects (direct or indirect) of different exposures is key in determining their contribution to observed disease outcomes. Lead exposure was shown to activate astrocytes as a potential mechanism of action when exploring neurotoxicity, but the authors suggest additionally investigating reactive oxygen species ROS) production [5]. *Aspergillus flavus* exposure in acute leukemia may lead to genetic and epigenetic alterations, which is hypothesized to be part of the mechanistic pathway [6]. It was further postulated that BPA serves as a marker for isoenzyme distribution patterns that disturb the endogenous metabolite environment, thus causing metabolic changes that favor disease development [7]. Certain viral or bacterial infections may be linked to disease development through molecular mimicry [8]. Many EDCs function as microbiome-disrupting chemicals as well, which may represent the causal pathway in disease development [9]. While flame retardants are known to disrupt endocrine pathways, insights into additional (carcinogenic) mechanisms are needed [10]. Further insights into molecular mechanisms will further propel potential preventative measures following exposure, targeted screening, and more personalized environmental medicine. On a more global scale, the accumulation of information regarding the impacts of chronic and cumulative exposure will inform legislative interventions to reduce exposures and improve global health.

## Data Availability

Not applicable.

## Abbreviations

The following abbreviations are used in this manuscript:

AD	Alzheimer’s disease
ADD	Attention-deficit disorder
ASDs	Autism spectrum disorders
BPA A	Bisphenol A
EBV	Epstein–Barr Virus
EDC	Endocrine-disrupting chemical
HHV-6A	Human herpesvirus 6A
PCOS	Polycystic ovary syndrome
PD	Parkinson’s disease
PVB19	Human parvovirus B19
SARS-CoV-2	Severe acute respiratory syndrome coronavirus 2

## References

[1] Wu H., Eckhardt C.M., Baccarelli A.A. (2023). Molecular mechanisms of environmental exposures and human disease. Nat. Rev. Genet..

[2] Fuller R., Landrigan P.J., Balakrishnan K., Bathan G., Bose-O’REilly S., Brauer M., Caravanos J., Chiles T., Cohen A., Corra L. (2022). Pollution and health: A progress update. Lancet Planet. Health.

[3] Prüss-Ustün A., van Deventer E., Mudu P., Campbell-Lendrum D., Vickers C., Ivanov I., Forastiere F., Gumy S., Dora C., Adair-Rohani H. (2019). Environmental risks and non-communicable diseases. BMJ.

[4] Peters A., Nawrot T.S., Baccarelli A.A. (2021). Hallmarks of environmental insults. Cell.

[5] Shvachiy L., Amaro-Leal Â., Machado F., Rocha I., Outeiro T.F., Geraldes V. (2024). Gender-Specific Effects on the Cardiorespiratory System and Neurotoxicity of Intermittent and Permanent Low-Level Lead Exposures. Biomedicines.

[6] Tebbi C.K., Sahakian E., Shah B., Yan J., Mediavilla-Varela M., Patel S. (2025). *Aspergillus flavus* with Mycovirus as an Etiologic Factor for Acute Leukemias in Susceptible Individuals: Evidence and Discussion. Biomedicines.

[7] Stein T.P. (2024). Does Bisphenol A (BPA) Exposure Cause Human Diseases?. Biomedicines.

[8] Cyna W., Wojciechowska A., Szybiak-Skora W., Lacka K. (2024). The Impact of Environmental Factors on the Development of Autoimmune Thyroiditis-Review. Biomedicines.

[9] Rosenfeld C.S. (2024). Should Pregnant Women Consume Probiotics to Combat Endocrine-Disrupting Chemical-Induced Health Risks to Their Unborn Offspring?. Biomedicines.

[10] Yeshoua B., Romero Castillo H., Monaghan M., van Gerwen M. (2024). A Review of the Association between Exposure to Flame Retardants and Thyroid Function. Biomedicines.

